# Risk factors for the growth of ground-glass nodules in the lungs: A systematic review and meta-analysis

**DOI:** 10.1016/j.clinsp.2025.100669

**Published:** 2025-05-07

**Authors:** Qianfang Yang, Fan Wang, Hongxin Cao

**Affiliations:** a Heilongjiang University of Traditional Chinese Medicine, China; b Harbin Medical University, China

**Keywords:** Ground-glass nodules, Lung cancer, Growth, Risk factors, Meta-analysis

## Abstract

**Objective:**

The growth of ground-glass nodules in the lungs is an independent risk factor for the occurrence of lung cancer, which provides the basic conditions for the occurrence of lung cancer. However, risk factors for the growth of ground-glass nodules in the lungs have not been fully identified. The purpose of this meta-analysis was to assess risk factors for the growth of ground-glass nodules in the lungs.

**Methods:**

Computerized searches of the electronic databases of PubMed, Web of Science, Cochrane Library and Scopus for published studies on risk factors for the growth of ground-glass nodules in the lungs. The search time limit is from the establishment of the database to March 2024. Two review authors independently searched the studies according to the inclusion and exclusion criteria, and the quality of the selected studies was evaluated using the Newcastle-Ottawa Scale (NOS), and RevMan 5.4 software was used for meta-analysis. This review is registered in the International Prospective Register of Systematic Reviews (PROSPERO) (identifier CRD42024499763).

**Results:**

Fourteen studies involving 2059 patients were included, and 14 statistically significant risk factors were identified. The results of meta-analysis showed that age (WMD = 4.61, 95 % CI [1.73∼7.49], *p* = 0.002), female (OR = 0.65, 95 % CI [0.51∼0.82], *p* = 0.0003), history of smoking (OR = 1.76, 95 % CI [1.07∼2.92], *p* = 0.03), history of malignancy (OR = 1.53, 95 % CI [1.16∼2.02], *p* = 0.003), lesion size (≥ 8 mm) (OR = 1.19, 95 % CI [1.12∼1.26], *p* < 0.00001), air bronchial sign (OR = 6.09, 95 % CI [3∼12.33], *p* < 0.00001), lobulation sign (OR = 2.3, 95 % CI [1.58∼3.36], *p* < 0.00001), spiculated sign (OR = 5.56, 95 % CI [1.39∼22.3], *p* = 0.02), vascular bundle sign [OR = 2.54, 95 % CI [1.85∼3.48], *p* < 0.00001), initial diameter (≥ 8 mm) (OR = 1.89, 95 % CI [1.34∼2.67], *p* = 0.0003), vacuolar sign (OR = 2.62, 95 % CI [1.46∼4.69], *p* = 0.001), solid nodules (OR = 4.6, 95 % CI [1.96∼10.79], *p* = 0.0005), solid components (OR = 13.77, 95 % CI [7.08∼26.78], *p* < 0.00001) and nodule roundness (OR = 2.85, 95 % CI [1.19∼6.81], *p* = 0.02) were risk factors for the growth of ground-glass nodules in the lungs. However, pleural adhesion (*p* = 0.47) and pleural retraction (*p* = 0.07) were not statistically significant.

**Conclusion:**

This systematic review and meta-analysis showed that there are many risk factors for the growth of ground-glass nodules in the lungs, and medical staff should identify the above risk factors as early as possible in clinical work and formulate targeted interventions for precise prevention.

## Highlights

• GGN is emerging as an important public health problem after COVID-19.
• The system evaluates the risk factors for GGN, enabling early identification of high-risk populations.
• Correctly identifying risk factors for GGN growth is clinically significant.

## Introduction

Ground-glass nodules of the lungs are a type of interstitial lung disease in which ground-glass nodular opacities occur within the lungs but are not sufficient to mask the underlying bronchial and vascular bundles, also known as ground-glass opacities of the lungs.^1^ According to the presence or absence of solid components in the nodules, GGNs can be further divided into pure Ground-Glass Nodules (pGGNs) and mixed Ground-Glass Nodules (mGGNs), where mGGNs contain solid and non-solid components, while pGGNs do not contain solid components.^2^ Studies have found that transient GGNs may disappear spontaneously, mainly due to inflammation or bleeding, while persistent GGNs are mostly associated with Atypical Adenomatous Hyperplasia (AAH), Adenocarcinoma In Situ (AIS), Minimally Invasive Adenocarcinoma (MIA) and Invasive Adenocarcinoma (IAC).^3^ Therefore, the correct identification of risk factors for the growth of GGNs is of great clinical significance and an important prerequisite for the development of interventions.

More and more studies have proven the close relationship between ground-glass nodules and lung cancer, and more attention has been paid to two-way screening of ground-glass nodules and lung cancer.^4^, ^5^, ^6^ However, current research evidence on risk factors for the growth of ground-glass nodules in the lungs is limited, and the assessment of risk factors for the growth of ground-glass nodules varies widely between studies.^7^, ^8^, ^9^ The aim of this study is to assess the risk factors for the growth of ground-glass nodules in the lungs by conducting a systematic literature review to provide a rationale for the early identification of high-risk populations.

## Method

This systematic review and meta-analysis was prospectively registered with the PROSPERO database of systematic reviews (CRD42024499763), and the results of this meta-analysis are reported in accordance with the Preferred Reporting Items for Systematic Reviews and Meta-Analysis (PRISMA) guidelines^10^ (S1 Checklist).

### Search strategy

First, the authors searched the PubMed, Web of Science, Cochrane Library and Scopus databases from inception to March 2024. Second, the reference lists of the included studies were examined as an additional check for potential studies that could be used in this review. The following keywords were used: (“Ground-glass nodules in the lungs” or “Ground glass nodules” or “Subsolid nodules”) and (“Growth” or “Increase”) and (“Risk factors” or “Influencing factors”). The specific search syntax, such as PubMed, can be found in S1 File.

### Eligibility criteria and exclusion criteria

Inclusion criteria: 1) Patients with GGNs confirmed by CT imaging, aged ≥18-years, 2) Risk factors for the growth of GGNs, 3) Cohort or case-control studies, 4) Articles must report Odd Ratios (ORs) or Hazard Ratios (HRs) with 95 % Confidence Intervals (95 % *CIs*) for relevant influencing factors, and 5) English in the language category.

Exclusion criteria: 1) Abstracts, letters, case reports, reviews, or non-clinical studies, 2) Studies with incomplete or unavailable data, 3) Duplicate or low-quality studies, and 4) Non-English studies.

### Study selection

Two investigators (QY and FW) read the title, abstract, and full text to identify the studies that met the inclusion criteria, and cross-checked the results of the included studies. The *Kappa* statistic was used to assess the reliability of data selection and selection between two investigators, and if there was disagreement, it was up to the third reviewer (HC) to decide whether to include them. Cohen suggested the *Kappa* result be interpreted as follows: values ≤ 0 as indicating no agreement 0.01 to 0.2 as none to slight, 0.2 to 0.4 as fair, 0.41 to 0.6 as moderate, 0.61 to 0.8 as substantial, and 0.81 to 1 as almost perfect agreement.^11^

### Data extraction

Two reviewers (QY and FW) independently evaluated the article and extracted the data. Study feature data were extracted for each article (first author; year of publication; country; study design; enrollment period; sample size; risk factors; NOS scores). During the data extraction process, any conflicts or ambiguities in the reporting method or results will be discussed with a third reviewer (HC) and resolved by consensus.

### Quality appraisal

The quality of the included studies was independently assessed by two review authors (QY and FW), and when disagreements were encountered, a third investigator (HC) was consulted and disagreements were resolved through discussion. All the studies included in this article were case-control studies, so the quality of the literature was evaluated by the NOS scale, which included 3 modules with a total of 8 items, with a full score of 9, 7∼9 for high-quality literature, 5∼6 for medium-quality literature, and ≤4 for low-quality literature.^12^

### Data analysis

Data analyses were conducted using RevMan 5.4 software, and STATA 17 software was used for Egger testing. The comprehensive meta-analysis allows for each of these different study outcomes to be flexibly entered into the model. *I*^2^ statistic was used to quantify the effect of heterogeneity. In the case of significant between-study heterogeneity (*I*^2^ value > 50 % and the p-value for Cochrane *Q* test < 0.1), the random-effects model was selected to compute the pooled estimate of risk factors. Otherwise, the fixed-effect model was used.^13^ To identify possible sources of heterogeneity, the authors performed sensitivity analysis. It used a fixed-effect model and a random-effects model to calculate the pooled effect size of the literature included in each risk factor, and the studies were excluded one by one by using the single-study exclusion method. Studies were deemed influential if their removal significantly modified the summary effect. For all tests, *p* ≤ 0.05 was deemed to be statistically significant.

#### Publication bias

Funnel charts were used to visually assess the existence of publication bias for the risk factors of ≥ 10 included papers, and the Egger test was used for statistical tests of asymmetric funnel plots. The Egger test was used to identify publication bias. If the 95 % Confidence Interval (95 % CI) of the intercept of the regression equation is found to contain 0 and *p* > 0.05, it indicates unbiased; otherwise, it is biased.^14^

## Result

### Search results and study characteristics

**Fig. 1:**
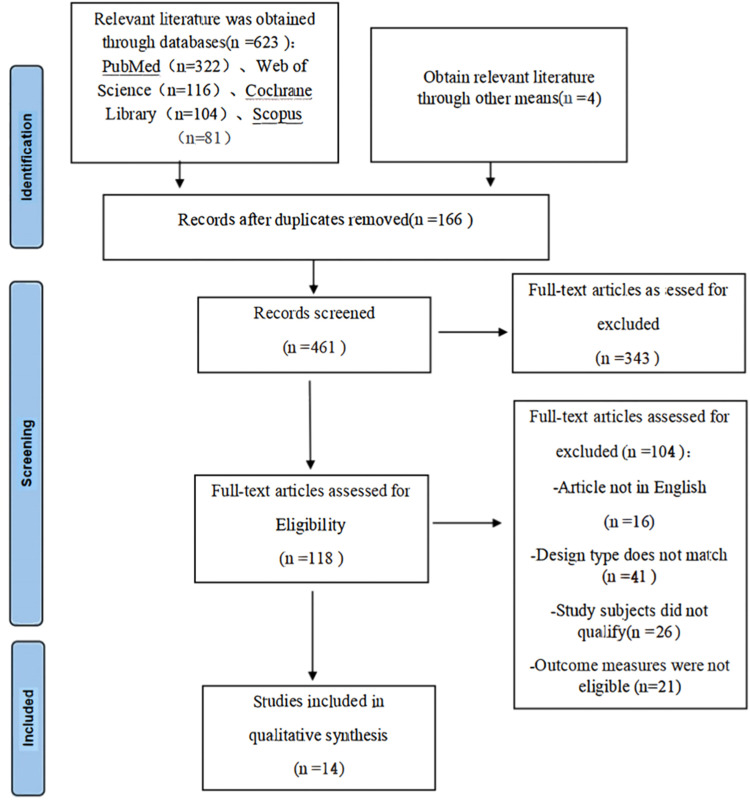
Process of study selection following the Preferred Reporting Items for Systematic Reviews and Meta-Analyses (PRISMA).

This search strategy (Fig. 1) identified a total of 627 unique records. Of these records, 14 studies met the inclusion/exclusion criteria and were included in the final meta-analysis.^15^, ^16^, ^17^, ^18^, ^19^, ^20^, ^21^, ^22^, ^23^, ^24^, ^25^, ^26^, ^27^, ^28^ A total of 2059 patients were pulmonary ground-glass nodule patients. All of these included studies were case-control studies and conducted from 1999 to 2022. The sample size of these studies ranged from 59 to 338. In terms of study country, 6 were from China, 5 from South Korea, and 3 from Japan. The details of the 14 studies included in the meta-analysis are shown in Table 1. The value of *Kappa* calculated for the various parameters extracted by the 2 investigators was 0.81 (*p* < 0.001), indicating an excellent degree of inter-investigator agreement.

**Table 1:** Characteristics of studies included in the meta-analysis.

Study	Country	Study design	Period	Sample size, N	Growth group, N	Non-growth group, N	Risk factors	Quality assessment tool	Scores
Xia et al. 202,0^15^	China	Case-control	2014∼2018	238	63	175	a, b, d, e, f, g, h, i, j, k, n, p	NOS	6
Yoon et al. 2019^16^	Korea	Case-control	2004∼2014	338	55	283	a, b, c, d, n	NOS	7
Qi et al. 201,9^17^	China	Case-control	2007∼2018	110	52	58	a, b, d, f, g, h, i, j, m	NOS	7
Xue et al. 2023^18^	China	Case-control	2017∼2021	116	54	62	a, b, c, d, f, g, h, i, k	NOS	7
Zhang et al. 2023^19^	China	Case-control	2007∼2017	306	163	143	b, c, d, g, i, j, k, l, m	NOS	6
Lee et al. 2019^20^	Korea	Case-control	2003∼2017	160	25	135	b, c, d, f, h, o	NOS	7
Lee et al. 2021^21^	Korea	Case-control	2009∼2018	82	33	43	a, b, c, d, e, o	NOS	7
Cho et al. 2016^22^	Korea	Case-control	2003∼2015	218	14	204	b, c, d, e, f, h, i, o	NOS	7
Tamura et al. 2014^23^	Japan	Case-control	2008∼2012	63	29	34	b, c, d, e	NOS	7
Shi et al. 2019^24^	China	Case-control	2011∼2012	59	16	43	l	NOS	7
Kobayashi et al. 2014^25^	Japan	Case-control	1999∼2013	86	34	52	b, c, d, l	NOS	6
Miyoshi et al. 2024^26^	Japan	Case-control	2006∼2016	82	35	47	n, o	NOS	7
Ryu et al. 2024^27^	Korea	Case-control	2008∼2022	69	27	42	f, n	NOS	7
He et al. 2022^28^	China	Case-control	2007∼2021	132	76	56	p	NOS	7

Note: *a* = Age; *b* = female; *c* = History of smoking (yes); *d* = History of malignancy; *e* = Lesion size (≥ 8 mm); *f* = Air bronchial sign; *g* = Lobulation sign; *h* = Spiculated sign; *i* = Pleural retraction; *j* = Pleural adhesion; *k* = Vascular bundle sign; *l* = Initial diameter (≥ 8 mm); *m* = Vacuolar sign; *n* = Solid nodules; *o* = Solid components; *p* = Nodule roundness; NOS = Newcastle-Ottawa Scale.

### Quality of methodology of the included studies

The quality evaluation of the literature showed that 3 articles^15^^,^^19^^,^^25^ were of moderate quality, 11 articles^16^, ^17^, ^18^^,^^20^, ^21^, ^22^, ^23^, ^24^^,^^26^, ^27^, ^28^ were of high quality, and no low-quality literature (Table S1).

### Synthetic results

#### Age

**Fig. 2:**
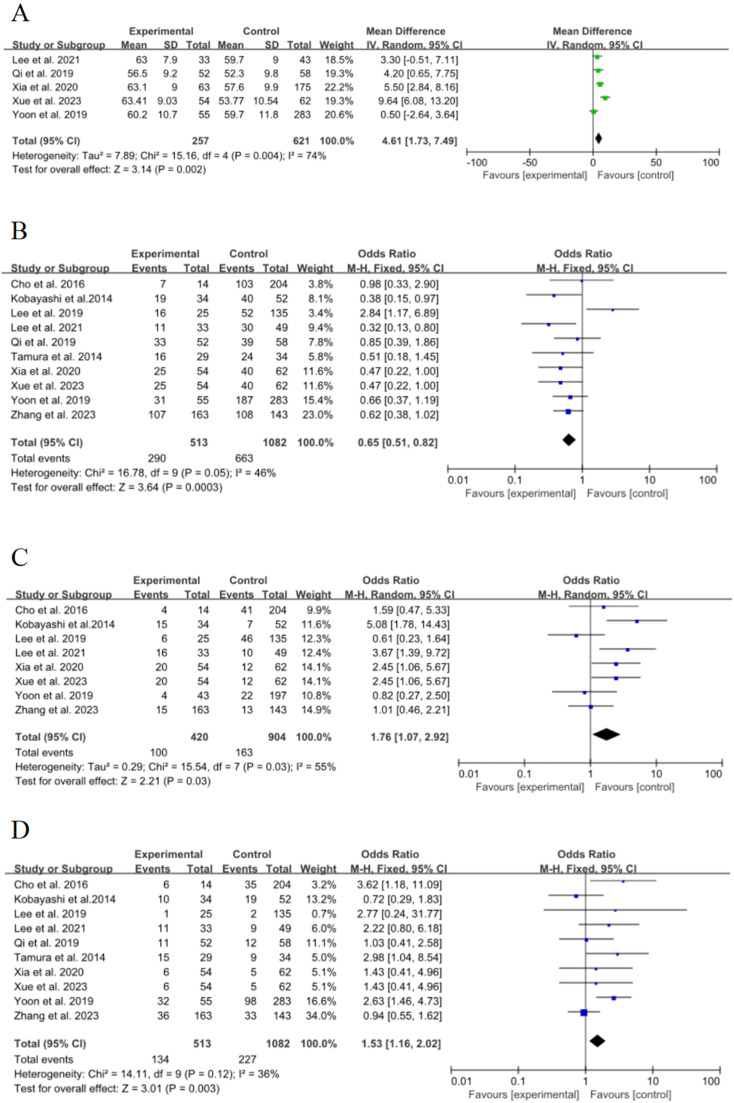
Forest plot of effects of risk factors on growth of ground-glass nodules in the lungs: (A) Age; (B) Female; (C) History of smoking; (D) History of malignancy.

Five of the 15 included studies^15^, ^16^, ^17^, ^18^^,^^21^ discussed the effect of age on the risk of poor growth of ground-glass nodules in the lungs, with statistical heterogeneity (*p* = 0.004, *I*^2^ = 74 %), and meta-analysis using a random-effects model. The results showed that age was an important risk factor for the growth of ground-glass nodules in the lungs, and the pooled effect was statistically significant (OR = 4.61, 95 % CI [1.73∼7.49], *p* = 0.002), as shown in Fig. 2A.

#### Female

Ten of the 15 included studies^15^, ^16^, ^17^, ^18^, ^19^, ^20^, ^21^, ^22^, ^23^^,^^25^ discussed the effect of women on the risk of poor growth of ground-glass nodules in the lungs, with no significant statistical heterogeneity between studies (*p* = 0.05, *I*^2^ = 46 %), and meta-analysis was performed using a fixed-effect model. The results showed that female was an important risk factor for the growth of ground-glass nodules in the lungs, and the pooled effect was statistically significant (OR = 0.65, 95 % CI [0.51∼0.82], *p* = 0.0003), as shown in Fig. 2B

### History of smoking

Eight of the 15 included studies^15^^,^^16^^,^^18^, ^19^, ^20^, ^21^, ^22^^,^^25^ discussed the effect of smoking history on the risk of poor growth of ground-glass nodules in the lungs, with statistical heterogeneity between studies (*p* = 0.03, *I*^2^ = 55 %), and meta-analysis was performed using a random-effects model. The results showed that smoking history was an important risk factor for the growth of ground-glass nodules in the lungs, and the combined effect was statistically significant (OR = 1.76, 95 % CI [1.07∼2.92], *p* = 0.03), as shown in Fig. 2C.

### History of malignancy

Ten of the 15 included studies^15^, ^16^, ^17^, ^18^, ^19^, ^20^, ^21^, ^22^, ^23^^,^^25^ discussed the effect of a history of malignancy on the risk of poor growth of ground-glass nodules in the lungs, with no significant statistical heterogeneity between studies (*p* = 0.12, *I*^2^ = 36 %), and meta-analysis was performed using a fixed-effect model. The results showed that the history of the malignant tumor was an important risk factor for the growth of ground-glass nodules in the lungs, and the combined effect was statistically significant (OR = 1.53, 95 % CI [1.16∼2.02], *p* = 0.003), as shown in Fig. 2D

### Lesion size (≥ 8 mm)

**Fig. 3:**
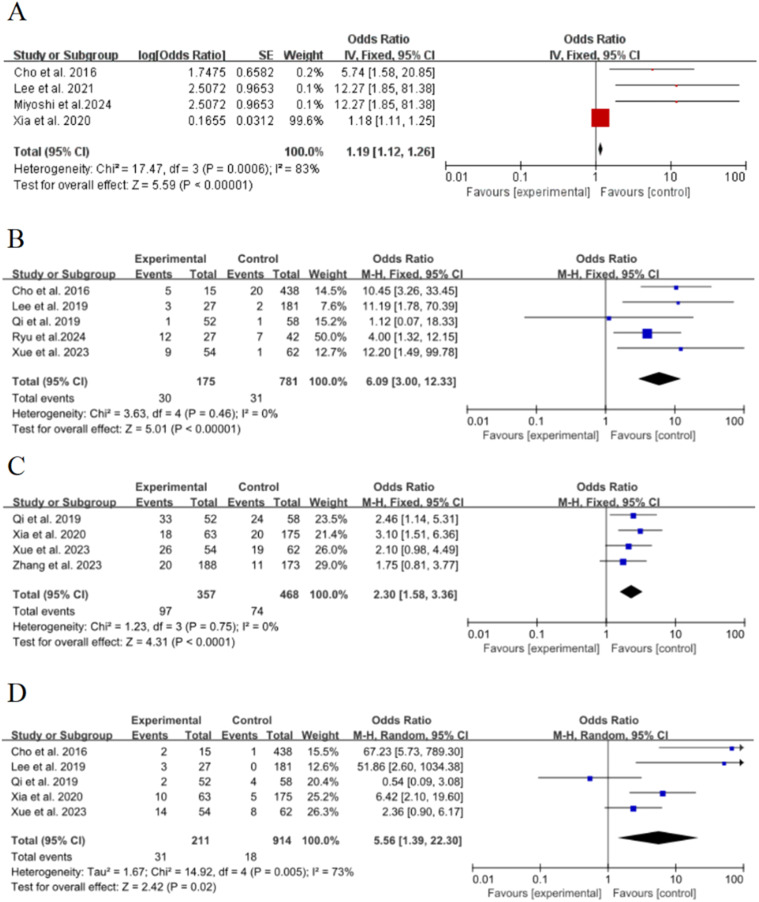
Forest plot of effects of risk factors on growth of ground-glass nodules in the lungs: (A) Lesion size (≥ 8 mm); (B) Air bronchial sign; (C) Lobulation sign; (D) Spiculated sign.

Four of the 15 included studies^15^^,^^21^^,^^22^^,^^26^ discussed the effect of lesion size (≥ 8 mm) on the risk of poor growth of ground-glass nodules in the lungs, with significant statistical heterogeneity between studies (*p* = 0.0006, *I*^2^ = 83 %), and meta-analysis was performed using a random-effects model. The results showed that lesion size (≥ 8 mm) was an important risk factor for the growth of ground-glass nodules in the lungs, and the combined effect was statistically significant (OR = 1.19, 95 % CI [1.12∼1.26], *p* < 0.00001), as shown in Fig. 3A.

### Air bronchial sign

Five of the 15 included studies^15^^,^^17^^,^^18^^,^^20^^,^^22^ discussed the effect of air bronchial signs on the risk of poor growth of ground-glass nodules in the lungs, with no statistical heterogeneity between studies (*p* = 0.46, *I*^2^ = 0 %), and a fixed-effect model was used for meta-analysis. The results showed that air bronchial sign was an important risk factor for the growth of ground-glass nodules in the lungs, and the combined effect was statistically significant (OR = 6.09, 95 % CI [3∼12.33], *p* < 0.00001), as shown in Fig. 3B

### Lobulation sign

Four of the 15 included studies^15^^,^^17^, ^18^, ^19^ discussed the effect of lobulation sign on the risk of poor growth of ground-glass nodules in the lungs, with no statistical heterogeneity between studies (*p* = 0.75, *I*^2^ = 0 %), and a fixed-effect model was used for meta-analysis. The results showed that lobulation sign was an important risk factor for the growth of ground-glass nodules in the lungs, and the combined effect was statistically significant (OR = 2.3, 95 % CI [1.58∼3.36], *p* < 0.00001), as shown in Fig. 3C.

### Spiculated sign

Five of the 15 included studies^15^^,^^17^^,^^18^^,^^20^^,^^22^ discussed the effect of needle margins on the risk of poor growth of ground-glass nodules in the lungs, with significant statistical heterogeneity between studies (*p* = 0.005, *I*^2^ = 73 %), and meta-analysis was performed using a random-effects model. The results showed that the needle-like margin was an important risk factor for the growth of ground-glass nodules in the lungs, and the combined effect was statistically significant (OR = 5.56, 95 % CI [1.39∼22.3], *p* = 0.02), as shown in Fig. 3D

### Pleural retraction

**Fig. 4:**
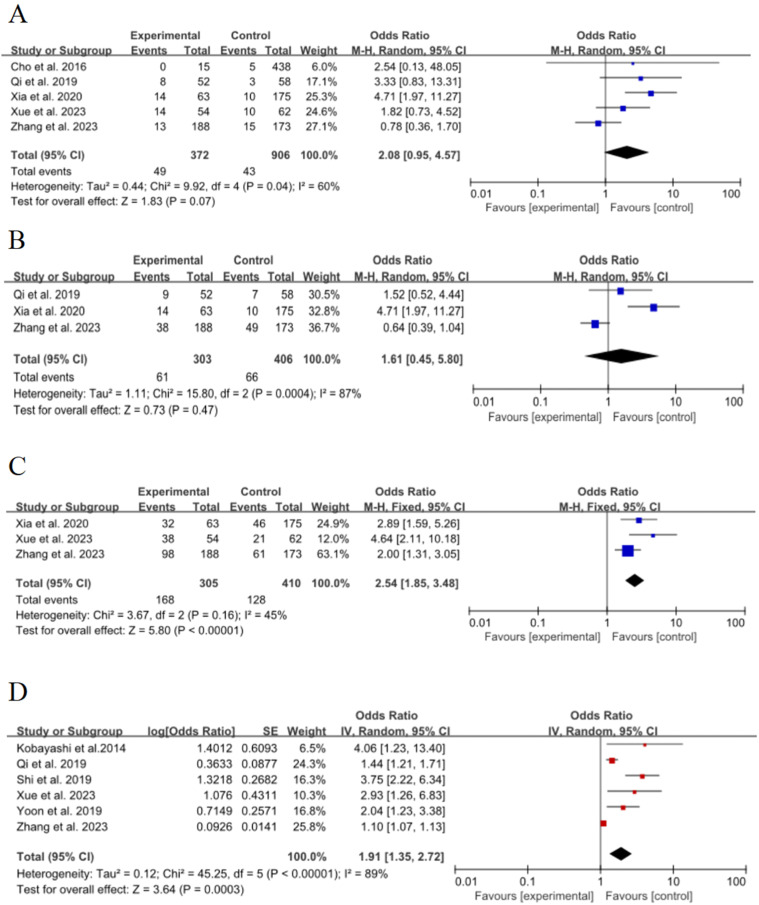
Forest plot of effects of risk factors on growth of ground-glass nodules in the lungs: (A) Pleural retraction; (B) Pleural adhesion; (C) Vascular bundle sign; (D) Initial diameter (≥ 8 mm).

Five of the 15 included studies^15^^,^^17^, ^18^, ^19^^,^^22^ discussed the effect of pleural depression on the risk of poor growth of ground-glass nodules in the lungs, with significant statistical heterogeneity between studies (*p* = 0.04, *I*^2^ = 60 %), and meta-analysis was performed using a random-effects model. The results showed that pleural depression was not a risk factor for the growth of ground-glass nodules in the lungs, and the combined effect was not statistically significant (OR = 2.08, 95 % CI [0.95∼4.57], *p* = 0.07), as shown in Fig. 4A.

### Pleural adhesion

Three of the 15 included studies^15^^,^^17^^,^^19^ discussed the effect of pleural adhesions on the risk of poor growth of ground-glass nodules in the lungs, with significant statistical heterogeneity between studies (*p* = 0.0004, *I*^2^ = 87 %), and meta-analysis was performed using a random-effects model. The results showed that pleural adhesion was not a risk factor for the growth of ground-glass nodules in the lungs, and the pooling effect was not statistically significant (OR = 1.61, 95 % CI [0.45∼5.8], *p* = 0.47), as shown in Fig. 4B

### Vascular bundle sign

Three of the 15 included studies^15^^,^^18^^,^^19^ discussed the effect of vascular bundle sign on the risk of poor growth of ground-glass nodules in the lungs, with no statistically significant heterogeneity between studies (*p* = 0.16, *I*^2^ = 45 %), and a fixed-effect model was used for meta-analysis. The results showed that vascular bundle sign was an important risk factor for the growth of ground-glass nodules in the lungs, and the combined effect was statistically significant (OR = 2.54, 95 % CI [1.85∼3.48], *p* < 0.00001), as shown in Fig. 4C.

### Initial diameter (≥ 8 mm)

Six of the 15 included studies^16^, ^17^, ^18^, ^19^^,^^24^^,^^25^ discussed the effect of initial diameter (≥ 8 mm) on the risk of poor growth of ground-glass nodules in the lungs, with significant statistical heterogeneity between studies (*p* < 0.00001, *I*^2^ = 89 %), and meta-analyses were performed using a random-effects model. The results showed that the diameter of the lesion (≥ 8 mm) was an important risk factor for the growth of ground-glass nodules in the lungs, and the combined effect was statistically significant (OR = 1.91, 95 % CI [1.35∼2.72], *p* = 0.0003), as shown in Fig. 4D

### Vacuolar sign

**Fig. 5:**
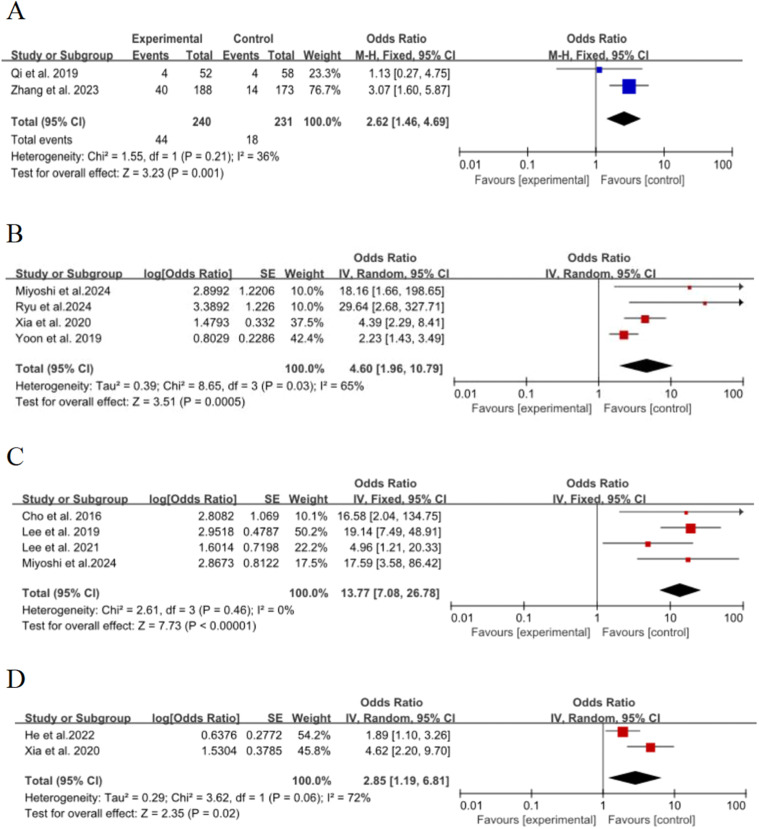
Forest plot of effects of risk factors on growth of ground-glass nodules in the lungs: (A) Vacuolar sign; (B) Solid nodules; (C) Solid components; (D) Nodule roundness.

Two of the 15 included studies^17^^,^^19^ discussed the effect of the vacuolar sign on the risk of poor growth of ground-glass nodules in the lungs, with no significant statistical heterogeneity between studies (*p* = 0.21, *I*^2^ = 36 %), and meta-analysis was performed using a fixed-effect model. The results showed that the vacuolar sign was an important risk factor for the growth of ground-glass nodules in the lungs, and the pooled effect was statistically significant (OR = 2.62, 95 % CI [1.46∼4.69], *p* = 0.001], as shown in Fig. 5A.

### Solid nodules

Four of the 15 included studies^15^^,^^16^^,^^26^^,^^27^ discussed the effect of the presence of partial solid nodules on the risk of poor growth of ground-glass nodules in the lungs, with significant statistical heterogeneity between studies (*p* = 0.03, *I*^2^ = 65 %), and meta-analysis was performed using a random-effects model. The results showed that the presence of partial solid nodules was an important risk factor for the growth of ground-glass nodules in the lungs, and the combined effect was statistically significant (OR = 4.6, 95 % CI [1.96∼10.79], *p* = 0.0005], as shown in Fig. 5B

### Solid components

Four of the 15 included studies^20^, ^21^, ^22^^,^^26^ discussed the effect of solid components on the risk of poor growth of ground-glass nodules in the lungs, with no statistical heterogeneity between studies (*p* = 0.46, *I*^2^ = 0 %), and a fixed-effect model was used for meta-analysis. The results showed that solid components were an important risk factor for the growth of ground-glass nodules in the lungs, and the combined effect was statistically significant (OR = 13.77, 95 % CI [7.08∼26.78], *p* < 0.00001), as shown in Fig. 5C.

### Nodule roundness

Two of the 15 included studies^15^^,^^29^ discussed the effect of nodule roundness on the risk of poor growth of ground-glass nodules in the lungs, with statistical heterogeneity between studies (*p* = 0.06, *I*^2^ = 72 %), and meta-analyses were performed using a random-effects model. The results showed that the roundness of the nodule was an important risk factor for the growth of ground-glass nodules in the lungs, and the combined effect was statistically significant (OR = 2.85, 95 % CI [1.19∼6.81], *p* = 0.02), as shown in Fig. 5D

### Sensitivity analysis

The results of sensitivity analysis showed that except for the vacuolar sign (*p* = 0.07) and pleural adhesion (*p* = 0.007), the combined OR values of other risk factors did not change differently after changing the effect model, indicating that the results of the meta-analysis were basically robust and reliable, as shown in Table 2. When Xue et al. 202,3^18^ were excluded, there was no significant statistical heterogeneity among the studies (*I*^2^ = 48 %, *p* = 0.12), and the results showed that age was a risk factor for the growth of ground-glass nodules in the lungs (WMD = 3.55, 95 % CI [1.95∼5.15], *p* < 0.0001). When Lee et al. 2019^20^ was excluded, there was no significant statistical heterogeneity among the studies (*I*^2^ = 43 %, *p* = 0.1), and the results showed that smoking history was a risk factor for the growth of ground-glass nodules in the lungs (OR = 2.04, 95 % CI [1.27∼3.29], *p* = 0.003). After Xia et al. 2020^15^ study was excluded, there was no statistical heterogeneity among the studies (*I*^2^ = 0 %, *p* = 0.73), and the results showed that lesion size (≥ 8 mm) was a risk factor for the growth of ground-glass nodules in the lungs (OR = 8.28, 95 % CI [3.27∼20.95], *p* < 0.00001). When Yoon et al. 2019^16^ were excluded, there was no significant statistical heterogeneity among the studies (*I*^2^ = 39 %, *p* = 0.19), and the results showed that the presence of partial solid nodules was a risk factor for the growth of ground-glass nodules in the lungs (OR = 5.44, 95 % CI [2.96∼9.98], *p* < 0.00001). Since only two studies^15^^,^^28^ on the roundness of nodules were included, a meta-analysis could not be performed when one study was excluded, so sensitivity analysis was no longer performed.

**Table 2:** Sensitivity analysis results.

Risk factors	Heterogeneity test results		Effect model	OR	95 % CI	*Z-*value	p-value
	*I*^2^	p-value					
Age	74 %	0.004	Fixed	4.57	3.12∼6.03	6.14	*p* < 0.00001^a^
Female	46 %	0.05	Random	0.64	0.46∼0.9	2.54	*p* = 0.01^a^
History of smoking (yes)	43 %	0.01	Random	2.04	1.27∼3.29	2.94	*p* = 0.003^a^
History of malignancy (yes)	36 %	0.12	Random	1.61	1.1∼2.35	2.47	*p* = 0.01^a^
Lesion size (≥ 8 mm)	0 %	0.73	Random	4.7	1.12∼19.62	2.12	*p* = 0.03^a^
Air bronchial sign	0 %	0.46	Random	6.61	3.37∼12.97	5.49	*p* < 0.00001^a^
Lobulation sign	0 %	0.75	Random	2.32	1.6∼3.39	4.39	*p* < 0.0001^a^
Spiculated sign	73 %	0.005	Fixed	3.39	1.87∼6.15	4.02	*p* < 0.0001^a^
Pleural retraction	87 %	0.0004	Fixed	1.07	0.73∼1.57	0.35	*p* = 0.73
Pleural adhesion	60 %	0.04	Fixed	1.85	1.18∼2.88	2.7	*p* = 0.007^a^
Vascular bundle sign	45 %	0.16	Random	2.73	1.73∼4.31	4.33	*p* < 0.0001^a^
Initial diameter (≥8 mm)	89 %	0.00001	Fixed	1.11	1.08∼1.14	7.65	*p* < 0.00001^a^
Vacuolar sign	36 %	0.21	Random	2.3	0.95∼5.61	1.84	*p* = 0.07
Solid nodules	39 %	0.19	Random	8.4	2.44∼28.87	3.38	*p* = 0.0007^a^
Solid components	0 %	0.46	Random	13.77	7.08∼26.78	7.73	*p* < 0.00001^a^
Nodule roundness	72 %	0.06	Fixed	2.58	1.67∼4.01	4.24	*p* < 0.0001^a^

a p < 0.05.

### Publication bias

**Fig. 6:**
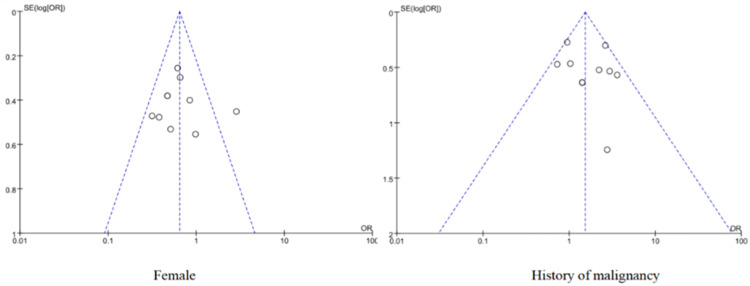
Female and history of malignancy risk factors published a funnel plot of bias.

The funnel plot shows a relatively uniform distribution of scatter plots for the included literature, as shown in Fig. 6. The results of Egger's test showed that were female (*t* = −0.56, *p* = 0.59) and had a history of malignancy (*t* = 1.48, *p* = 0.17), indicating that there was little possibility of publication bias (Figs. S1 and S2).

## Discussion

In recent years, with the improvement of people's attention to health and the development and progress of CT imaging technology, the detection rate of GGNs has continued to increase, and the high malignant tendency caused by the growth of pulmonary ground-glass nodules has attracted the attention of scholars at home and abroad. The authors searched databases and found few reports of risk factors for the growth of ground-glass nodules in the lungs, with only two systematic review reports and risk factor results similar to ours.^29^^,^^30^ A total of 14 case-control studies were included in this study, and the quality evaluation results showed that 11 high-quality literature, 3 medium-quality literature, and no low-quality literature, and the quality of the literature met the requirements. In addition, the inclusion and exclusion criteria of the respondents and the risk factors for the growth of ground-glass nodules in the lungs were clearly stated in all the literature, and the statistical methods used were correct. Therefore, this meta-analysis has a high degree of confidence.

The results of the meta-analysis showed that age (WMD = 4.61), female (OR = 0.65), smoking history (OR = 1.76), malignancy history (OR = 1.53), lesion size (≥ 8 mm) (OR = 1.19), air bronchial sign (OR = 6.09), lobulation sign (OR = 2.3), spiculated sign (OR = 5.56), vascular bundle sign (OR = 2.54), lesion diameter (≥ 8 mm) (OR = 1.89), vacuolar sign (OR = 2.62), presence of partial solid nodules (OR = 4.6), presence of solid components (OR = 13.77) and roundness of nodules (OR = 2.85) were risk factors for the growth of ground-glass nodules in the lungs. However, pleural adhesion (*p* = 0.47) and pleural retraction (*p* = 0.07) were not statistically significant.

Furthermore, lesion size, history of smoking, air-bronchial sign, lobulation sign, spiculated sign, vascular bundle sign, vacuolar sign, and solid nodules, results were highly heterogeneous, and the authors found that after a sensitivity analysis one by one, the reasons for the high heterogeneity may be related to factors such as different disease cycles, differences in sample size, and disease severity in the included patients. For example, Lee et al. 2019^20^ included patients with disease cycles from 2003∼2017, which spanned a long period of time and was significantly different from other study cycles, so when this study was excluded, there was no significant statistical heterogeneity between the included smoking history studies (*I*^2^ = 43 %, *p* = 0.1).

With age, lung tissue will undergo cell enlargement, protein synthesis, and mitochondrial oxidative phosphorylation levels, resulting in morphological changes in lung tissue.^31^ Zhang^32^ showed that age is a key risk factor for the growth and malignant transformation of GGNs, and the detection rate of lung cancer gradually increased with the age of the participants. In addition, a meta-analysis showed that 11 of the 20 included studies reported an overall positive association between older age and the risk of pulmonary nodules.^33^

Smoking is an important risk factor for the development of ground-glass nodules in the lungs. Studies have shown that smoking may induce the production of pulmonary nodules by affecting DNA transcription and translation processes or by directly damaging DNA.^34^^,^^35^ In addition, smoking history is an independent risk factor for lung cancer, with 68.04 % of male lung cancer patients and 26.51 % of nonsmokers attributable to passive smoking.^36^^,^^37^

Gender is a key risk factor for the development of pulmonary nodules, and there is a significant correlation between the occurrence of pulmonary nodules, and the incidence is higher in women than in men. A retrospective analysis of the pathogenesis and associated factors of pulmonary nodules found that the proportion of women with pulmonary nodules was higher, at approximately 36.5 %.^38^

Several studies have shown that a history of malignancy correlates with GGN growth.^39^, ^40^, ^41^ Shewale^42^ systematically reviewed 210 patients with GGN with a history of lung cancer and demonstrated that patients with a history of lung adenocarcinoma were6.85 times more likely to have GGN growth than patients with other lung cancer subtypes.

Air bronchial sign refers to the presence of air-containing lung tissue and dilated small bronchi that have not yet been occupied by tumor tissue in the lesion, and the imaging is mostly manifested as a gas density shadow of < 5 mm in diameter, which is common in lung cancer.^43^ Kou^44^ collected CT images from 132 patients with GGNs and found that the signs of bronchial inflation in IAC were much higher than those in AAH/AIS/MIA. The passage of blood vessels through the lesion tissue is called the vascular bundle sign, and the highly aggressive lesion tissue has a rich blood supply and is conducive to the growth of the lesion tissue.^45^ Studies have found that^46^ most of the blood vessels in the nodules of malignant GGNs are abnormal, which may be related to the aggressiveness of the tumor tissue, and because the tumor tissue is hyperbole, it needs more blood to maintain growth, resulting in thickening and dilation of blood vessels.

An increase in the density within GGNs (an increase in solid components) is positively correlated with the degree of malignancy.^47^ The risk of malignancy was found to be higher with GGNs than with solid nodules, with mGGNs having the highest incidence of malignancy (63 %), followed by pGGNs (18 %), and solid nodules having a malignant incidence of only 7 %.^48^ The diameter of the nodule is the main reference factor for the malignant growth of GGN. Studies have shown that for lung nodules with a diameter of < 5 mm, the risk of malignant tumors is 0.4 %, while the risk of malignancy of pulmonary nodules with a diameter of 5∼10 mm is 1.3 %; When the nodule diameter is 10∼20 mm, the risk of malignancy is 33 %, and when the nodule diameter is > 20 mm, the risk of malignancy increases to 60 %.^49^

The advantage of the systematic reviews and meta-analyses is that they fully follow PRISMA claims and that the review methods are registered. These findings reveal risk factors for the growth of ground-glass nodules in the lungs and can be further used for cost-benefit analysis and cost-utility analysis of lung cancer screening programs. However, the present study has some limitations that are worth discussing. First, the authors included only English-language studies in this meta-analysis, and the selection of participants and sample size were insufficient and there was some bias. Second, there was significant heterogeneity between the included studies, which may be related to factors such as different disease cycles, differences in sample size, and disease severity in the included patients. Third, the studies included in the literature were all case-control studies, and the strength of the evidence for such studies was weak. Finally, in most of the included studies, there was a lack of data on risk factors for spiculated sign and initial diameter (≥8 mm), which limited the ability to perform subgroup analyses to explore sources of heterogeneity.

## Conclusion

Effective intervention against the above risk factors can reduce the risk of pulmonary ground-glass nodule growth and improve the clinical prognosis of patients with pulmonary ground-glass nodules. However, due to the limitations of this study, the authors hope to have more high-quality, prospective, large-sample, and multicenter studies in future studies to support these findings.

## Funding

This work is not supported by funding.

## CRediT authorship contribution statement

**Qianfang Yang:** Methodology, Writing – review & editing. **Fan Wang:** Methodology, Writing – review & editing. **Hongxin Cao:** Validation, Writing – review & editing.

## Declaration of competing interest

The authors declare no conflicts of interest.

## Appendix Supplementary materials

Image, application 1
